# Breast cancer in patients under 40 years: an 11-year retrospective cohort analysis

**DOI:** 10.1007/s00404-025-08031-5

**Published:** 2025-05-15

**Authors:** Franziska Ganster, Simone Schrodi, Michael Braun, Christina Seifert, Sven Mahner, Thomas Kolben, Rachel Wuerstlein, Nadia Harbeck, Maximiliane Burgmann

**Affiliations:** 1 Department of Gynecology and Obstetrics and Comprehensive Cancer Center Munich LMU, BZKF Breast Center, LMU University Hospital, Marchioninistr. 15, 81377 Munich, Germany; 2 Bavarian Cancer Registry, Bavarian Food and Health Authority (LGL), Munich, Germany; 3 Department of Gynecology, Breast Center, Red Cross Hospital, Munich, Germany

**Keywords:** Premenopausal patients, Breast cancer, Tumorbiology, Survival, Time trend

## Abstract

**Introduction:**

The number of young breast cancer (BC) patients is increasing in both high- and low-income countries. It is known that this population is at risk for more aggressive tumor phenotypes, larger tumor size at diagnosis and poorer prognosis. It is the aim of this population-based analysis to identify trends of therapy, tumor biology and prognosis during a period of 11 years in young patients under the age of 40.

**Methods:**

In this analysis, data of young BC patients (< 40 years) from two breast centers were collected and analysed. The focus was a summary of data regarding tumor phenotypes, treatment, and survival in young BC patients.

**Results:**

Out of 11,954 patients with invasive BC who were eligible to the analysis, 781 (6.5%) were younger than 40 years at diagnosis and met the inclusion criteria. The predominant biological subtypes were Luminal B-like (HER2−) and Luminal-A-like, 62.3% were diagnosed with pN0. Noticeably low rates for endocrine therapy and higher rates for chemotherapy could be observed. 10-year overall survival was 87% for the whole cohort. Luminal-B-like (HER2−) and Triple negative tumors had worse outcomes as opposed to the other subtypes.

**Conclusion:**

As a conclusion, this 11-year analysis provides valuable insights into the clinical characteristics and treatment outcomes of young breast cancer patients under 40 years of age. The analysis highlights clear outcome differences according to the tumor subtype. These findings underscore the need for personalized treatment approaches and continued follow-up to optimize outcomes for young BC patients.

## What does this study add to the clinical work

**Table:** 

In young BC patients under 40 years the predominant biological subtypes were Luminal B-like (HER2-) and Luminal-A-like. They underwent low rates for endocrine therapy and higher rates for chemotherapy while the 10-year overall survival was 87% for the whole cohort and subtypes.	

## Introduction

Breast cancer (BC) is a common health problem affecting women worldwide, with the impact on younger people being increasingly recognised [1]. The International Consensus Conference for Breast Cancer in Young Women (BCY1) in 2012 and the European Society of Breast Cancer Specialists (EUSOMA) in 2013 have collectively defined"young patients"as those diagnosed with breast cancer under an age of 40 [2, 3]. By following this definition, we focus the unique challenges and considerations associated with breast cancer in this demographic stratum. 

Despite their representing a smaller proportion of breast cancer cases as compared with older women, young patients comprise a significant portion of breast cancer diagnoses, with approximately 5–10% in high-income countries and 55% in low- and middle-income countries [4]. Moreover, there has been a notable increase in breast cancer incidence both among Caucasian women and young black women in the United States [5–7].

The age at diagnosis is not the only factor which contributes to the distinctive characteristics of breast cancer in young women. Premenopausal women are known show more aggressive tumor phenotypes, often characterized by larger tumor sizes and higher grade tumors, leading to poorer prognosis compared with older counterparts [8–10]. This outcome disparity results from various factors, including differences in tumor biology, suboptimal endocrine treatment, and decreased adherence to adjuvant endocrine therapy [9]. As a result, young breast cancer patients often require more intensive treatment regimens with the aim of maximizing therapeutic benefits while minimizing long-term toxicities [10].

In awareness of the multifaceted requirements of young breast cancer patients, the International Consensus Conference for Breast Cancer in Young Women 2020 (BCY5) stressed a comprehensive care provided by specialized breast centers [11]. The centers not only offer surgical and systemic treatments, but also provide essential psychosocial support, genetic counselling, and fertility preservation services which are of high importance to young BC patients [12–15]. Furthermore, young women are encouraged to undergo consultations addressing lifestyle factors such as body mass index, alcohol consumption, physical activity, and smoking habits [12, 16–20].

In the light of the increasing number of young women diagnosed with breast cancer, there is a pressing need to evaluate trends in tumor biology, therapy modalities, and survival in premenopausal breast cancer patients. By examining these evolving patterns, we aim to gain insights into the current landscape of breast cancer care for young women and identify areas for further improvement and intervention. This analysis will provide valuable evidence and enhance the management and outcomes of breast cancer in this vulnerable population. Through a comprehensive analysis of tumor characteristics, treatment approaches, and survival, we aim to inform about strategies that optimize care and support for young women confronted with a breast cancer diagnosis.

## Methods

### Data collection

Data were provided and analysed by the former Munich Cancer Registry (MCR) of the Munich Tumor Center (TZM). The MCR was a population-based clinical cancer registry of Bavaria/Southern Germany with a total catchment of about 5 million inhabitants. In this catchment area, all pathology reports were required to be submitted to the cancer registry. In parallel, patient demographics, treatment, and follow-up information were reported from clinicians. Additionally, the life status was maintained systematically through death certificates. All data were documented according to the guidelines of the International Agency for Research in Cancer. Due to law changes, since 2018, data are reported to and documented by the Bavarian Cancer registry, which is part of the Bavarian Health and Food Safety Authority (LGL).

### Cohort selection

**Fig. 1:**
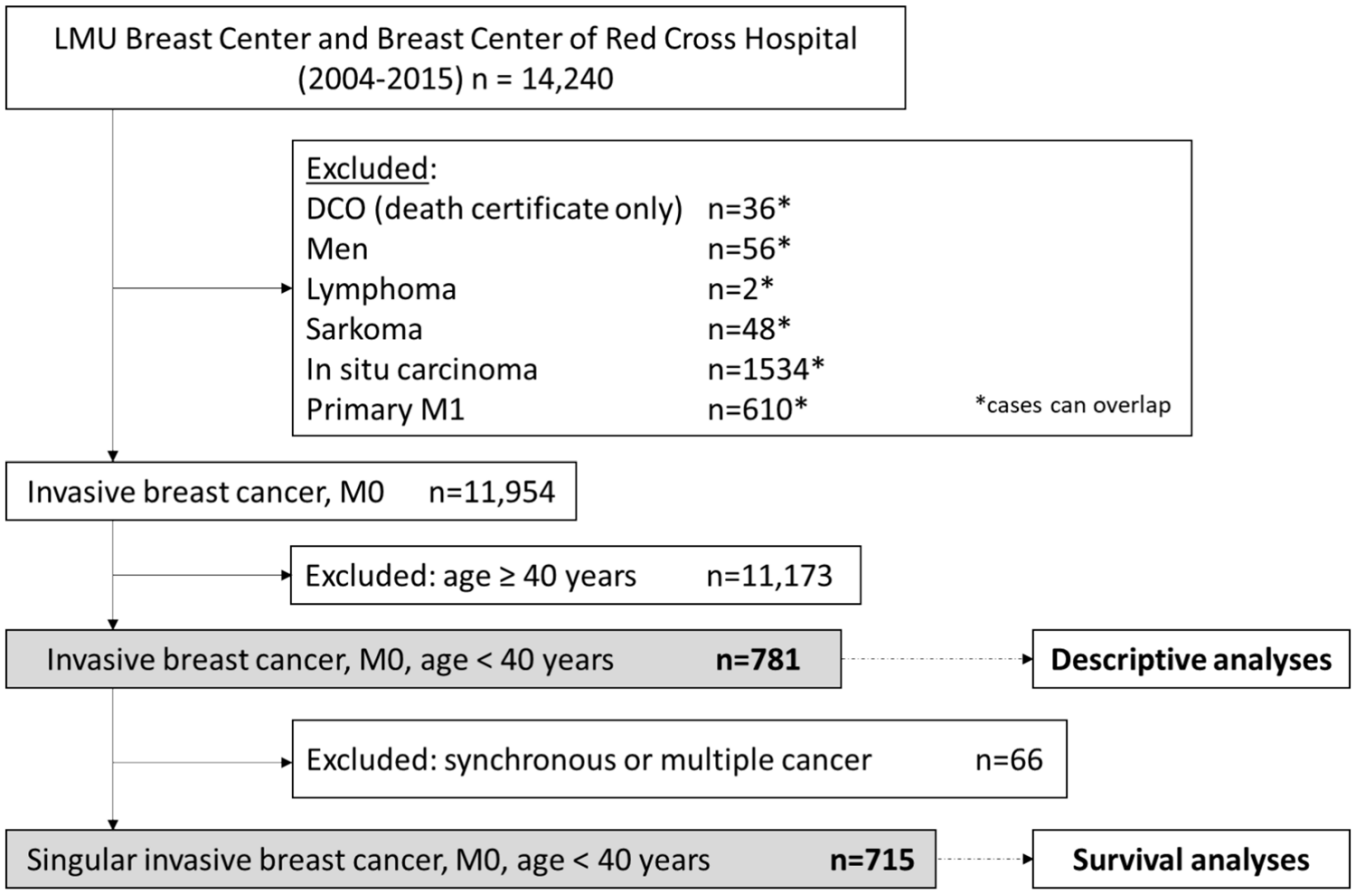
Cohort selection

From the 11,954 invasive breast cancer patients with diagnosis between 2004 and 2015 in either the LMU Breast Center or the Breast Center of Red Cross Hospital, 781 young patients (< 40 years) were included. Excluded were male patients, histology of lymphoma or sarcoma or non-invasive histology, and patients with primary metastasis (M1). In the survival analysis, patients with evidence of another previous or synchronous malignant tumor were additionally excluded to eliminate any overlapping effects (Fig.  1).

### Definition of variables

Tumors were classified according to the TNM classification of malignant tumors (8 Edition) [21]. Since molecular subtypes are not available in the cancer registry, subtypes were coded due to an alternative classification using estrogen receptor (ER), progesterone receptor (PR), HER2 expression, Ki-67, and Grade. Accordingly, five subgroups were distinguished: “Luminal A-like” (HER2−, ER and/or PR+, Ki-67<10 or Grade 1/2); “Luminal B-like (HER2−)” (HER2−, ER and/or PR+, Ki-67≥10 or Grade 3); “Luminal B-like (HER2+)” (HER2+, ER and/or PR+); “HER2-like non-luminal” (HER2+, ER−, PR−); and “Triple negative” (HER2−, ER−, PR−). ER and PR were regarded as positive, if at least 1.0% of the cells were positive. HER2 expression was evaluated based on IHC and in situ hybridization (FISH/chromogenic in situ hybridization) according to the ASCO/CAP guideline [22].

### Statistical analysis

The MCR organized data in an Oracle database. Statistical analyses were conducted using SAS (version 9.4; SAS Institute, Cary, NC). The prognostic factors and therapies were analysed using descriptive statistics. The percentages of the presented subcategories were related to the sum of available data of each variable, while missing data were not taken into account.

In the survival analysis, overall survival (OS) and relative survival (RS) was computed using the Kaplan–Meier Method. RS was computed by calculating the ratio of the observed survival rate to the expected survival rate. The expected survival time of age-matched individuals was calculated using life tables for the German population using the Ederer II method [23]. RS can be interpreted as survival from cancer after correcting for other causes of death, therefore RS was used to estimate cancer-specific survival. Additionally, time to local recurrence (TTLR) and time to metastasis (TTM) were used as endpoints in this analysis because they are surrogate parameters for survival.

## Results

### Prognostic factors and therapies

In the period from 2004 to 2015, 11,954 patients had been diagnosed and treated with invasive BC at LMU Breast Center and of Munich Red Cross Hospital and were eligible to the analysis. Out of these, 781 (6.5%) patients were under the age of 40 years and met the inclusion criteria.

Among young BC patients, the majority (*n* = 272, 60.6%) was diagnosed with BC stage pT1, followed by 162 patients with pT2 (36.1%), 14 patients with pT3 (3.1%) and one patient with pT4 (0.2%).

Regarding the nodal state in young BC patients, more than 50% were diagnosed with pN0 (*n* = 466; 64.2%), followed by pN+ (*n* = 257; 35.4%) and only 3 patients with pNX (0.4%). The majority of young BC patients was diagnosed with Grade 3 (*n*=362, 48.0%) followed by Grade 2 (*n* = 344, 45.6%) and G1 (*n* = 49, 6.5%).

Regarding the estrogen and progesterone receptor state, young BC patients seemed to have increased numbers of ER positive BC (*n*=529, 69.2% of young BC) and PR positive BC (*n* = 506; 66.3% of young BC). HER2 state was predominantly negative in young BC (*n* = 564; 74.4%).

Most of the young BC patients were diagnosed with biological subtype Luminal-B-like (HER2−) BC (ER positive, PR positive and HER 2 negative). Table 1 sums up the data collected between 2004 and 2015.

**Table 1:** Tumor classification, tumor biology and treatment of breast cancer patients < 40 years (*n* = 781)

Tumor classification	
T	(*n* = 449; 332 missing data)
T1	272 (60.6%)
T2	162 (36.1%)
T3	14 (3.1%)
T4	1 (0.2%)
N	(*n* = 726; 55 missing data)
N0	466 (64.2%)
N +	257 (35.4%)
Nx	3 (0.4%)
Grading	(*n* = 755; 26 missing data)
G1	49 (6.5%)
G2	344 (45.6%)
G3	362 (48.0%)
Tumor biology	
ER	(*n* = 764; 17 missing data)
Positive	529 (69.2%)
Negative	235 (30.8%)
PR	(*n* = 763; 18 missing data)
Positive	506 (66.3%)
Negative	257 (33.7)
ER/PR	(*n* = 764; 17 missing data)
Positive	568 (74.4%)
Negative	196 (25.6%)
HER2-Status^a^	(*n* = 758; 23 missing data)
Positive	172 (22.7%)
Negative	564 (74.4%)
HER2 (2 +)	22 (2.9%)
Subtype	(*n* = 733; 48 missing data)
Luminal-A like	194 (26.5%)
Luminal-B like (HER2)	225 (30.7%)
Luminal-B like (HER2 +)	125 (17.1%)
HER2 +/non-luminal	47 (6.4%)
Triple negative	142 (19.4%)
Local therapy	
Breast surgery	(*n* = 751; 30 missing data)
No surgery	12 (1.6%)
Breast conserving surgery	483 (64.3%)
Mastectomy	256 (34.1%)
Axillary surgery	(*n* = 781)
No axillary surgery	26 (3.3%)
Locally axillary dissection (LAD)	204 (26.1%)
SLNB + LAD	166 (21.3%)
SLNB only	356 (45.6%)
Other axillary surgery	29 (3.7%)
Radiation	(*n* = 483; 298 missing data)
Yes	386 (79.9%)
No	97 (20.1%)
Radiation after mastectomy	(*n* = 256; 525 missing data)
Yes	122 (47.7%)
No	134 (52.3%)
Systemic therapy^b^	
Chemotherapy, endocrine therapy	(*n* = 781)
No systemic therapy	70 (9.0%)
Chemotherapy only	229 (29.3%)
Endocrine therapy only	145 (18.6%)
Chemotherapy and endocrine therapy	337 (43.2%)
Targeted therapy	(*n* = 172; 609 missing data)
Yes	131 (76.2%)
No	41 (23.8%)

^a^HER 2 positive: IHC Score = 3 or IHC Score = 2 and FISH-Test positive

HER 2 negative: IHC Score = 1 or IHC Score = 2 and FISH-Test negative

^b^Therapy Yes: recommended, started, completed. Therapy No: contraindicated, rejected by the patient, not completed

**Fig. 2:**
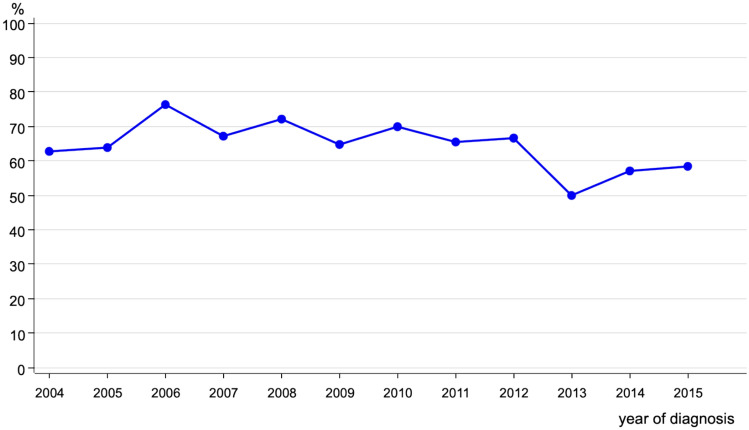
Trend of breast conserving surgery in breast cancer patients < 40 years (*n* = 483)

Regarding the treatment, 739 of young BC patients underwent surgery. The major part of this group (*n* = 483; 64.3%) got a breast conserving surgery. Summarized in Fig. 2 is the trend of breast conserving surgery over the period of the analysis. We can see stable rates from 2004 to 2012 and from 2013 onwards a decrease in numbers.

**Fig. 3:**
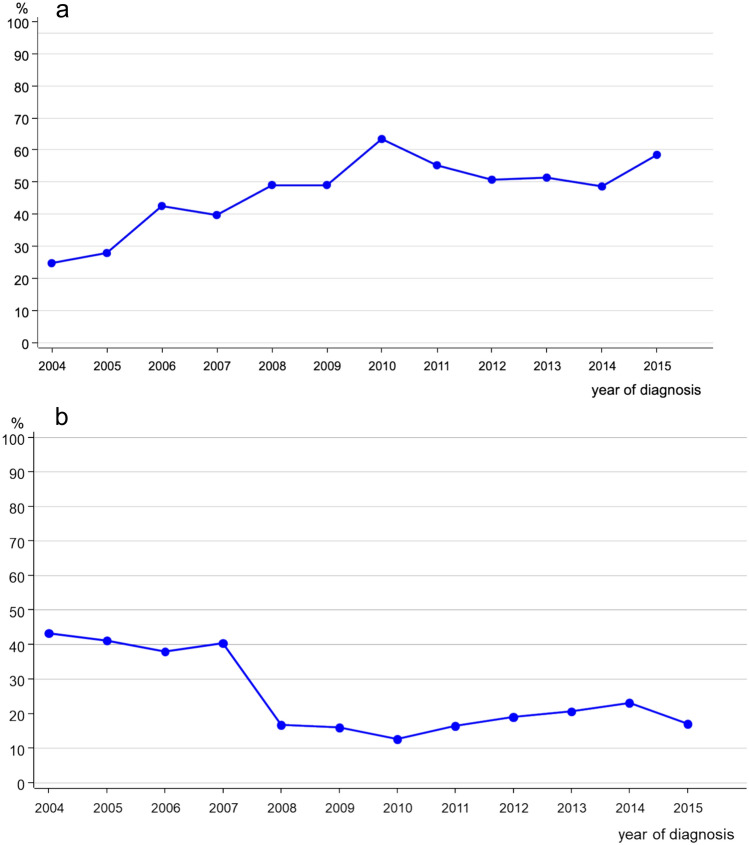
**a** Trend of sentinel lymph node biopsy alone (SLNB) in breast cancer patients < 40 years (*n* = 356). **b **Locally axillary dissection (LAD) in breast cancer patients < 40 years (*n* = 204)

The numbers regarding axillary dissection, demonstrated in Fig.  3a and b, show that most of young BC patients got a sentinel lymph node biopsy (SLNB) only (*n* = 356, 45.6%), showing a significant increase over time, then stable at a high level at 50–60% from 2010 onwards, while the number of LADs fell abruptly from 2008 onwards.

In addition to the surgical therapy, for 48% of the patients with BCS, a radiation therapy was documented (*n* = 386; 47.7%).

**Fig. 4:**
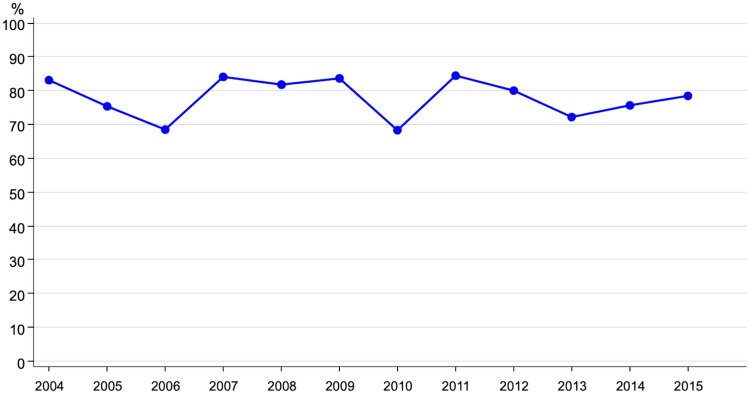
Trend of chemotherapy in young breast cancer patients (*n* = 566)

**Fig. 5:**
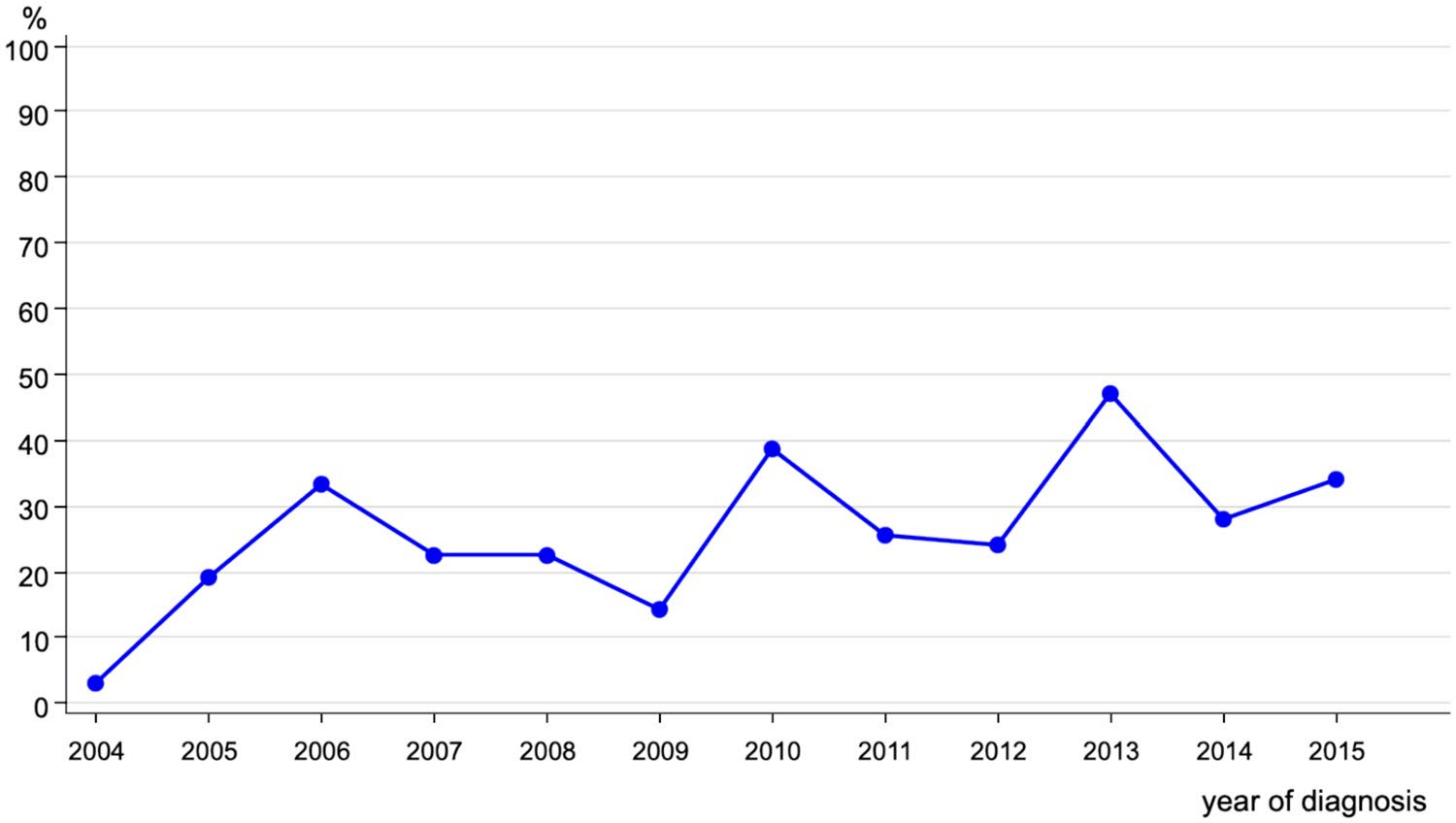
Trend of endocrine therapy for patients < 40 years (HR +, *n* = 568)

Regarding an additional systemic treatment 43.2% (*n* = 337) of the young patients received both adjuvant chemotherapy (CT) and endocrine therapy (ET), 29.3% (*n* = 229) CT only and 18.6% (*n* = 145) ET only (see Table 1). Figures 4 and 5sum up the CT and ET trends. We can see stable rates regarding CT, regarding ET with HR + increase over time.

### Survival analysis

**Fig. 6:**
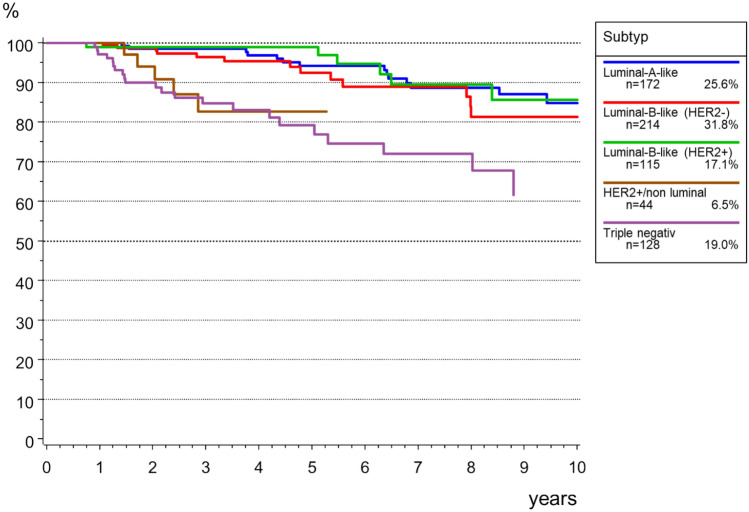
Overall survival in breast cancer patients < 40 years (M0, *n* = 673)

The overall survival (OS) of the 673 young patients without primary metastasis (M0) is shown in Fig.  6. The 5-year OS was 90.9% and after 10 years, 79.3% were still alive. Due to the young age, the relative survival (RS) was comparable with 90.9% and 79.0%, respectively.

**Fig. 7:**
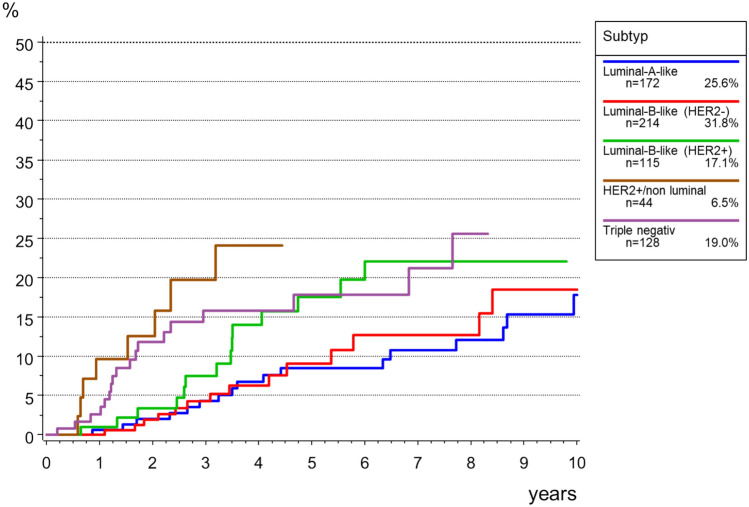
Cumulative incidence for time to local recurrence in in breast cancer patients < 40 years (M0, *n* = 673)

Local recurrence rate was 13.4% after 5 years, and 23.1% after 10 years. Figure 7 shows the data for time to local recurrence depending on the biological subtypes. The highest incidence for local recurrence in young BC patients was among those with triple negative BC. However, later local recurrence was higher in HER2 + non-luminal BC patients.

The cumulative incidence of the time to distant metastasis (TTM) for young M0 patients was 16.3 (95% CI 13.1–19.8) after 5 years and 26.1 (95% CI 21.1–31.4) after 10 years.

**Fig. 8:**
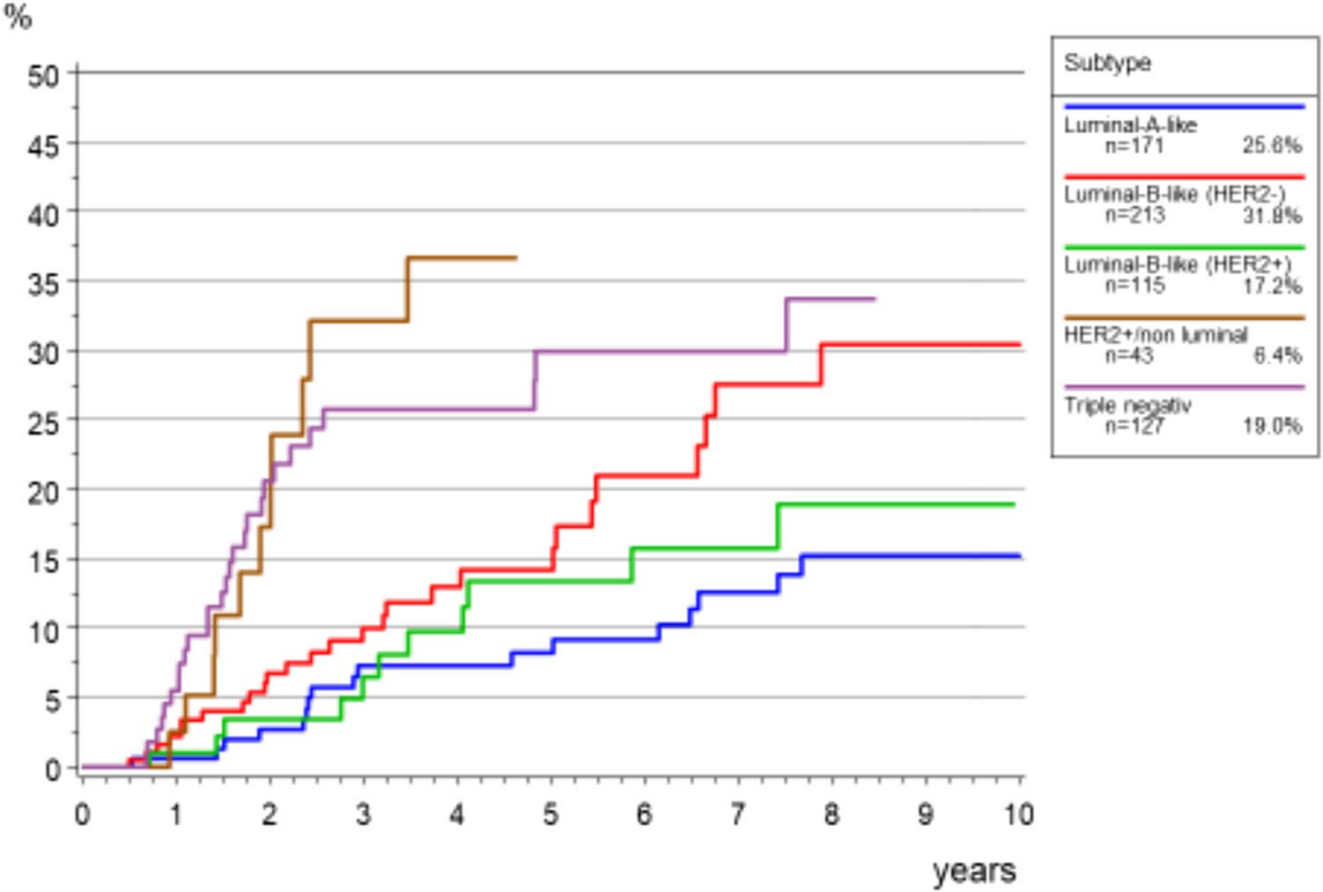
Cumulative incidence of time to distant metastasis (TTM) according to subtype for young patients with breast cancer (M0, *n* = 709)

Figure  8 shows the TTM rates according to the biological subtype. Tumors of the subgroups HER2 + non luminal and Triple negative showed earlier and more frequent diagnosis of distant metastases as compared to the other subgroups (see Fig.  9)

## Discussion

Our analysis provides valuable insights into the clinicopathological characteristics, modalities and outcomes of breast cancer (BC) treatment in young women aged under 40 years. There is a substantial proportion of BC diagnoses occurring in young women, comprising 6.5% of the total BC cases over the period analysed. This underscores the importance of understanding and addressing BC in younger individuals 

Previous studies have shown that younger women are more likely to have larger tumors and higher grade tumors, suggesting a more aggressive cancer biology [8–10, 24–26]. We were able to confirm that young BC patients develop tumors with higher grade (G3 in 48.0%), but not tumors with higher stage, since in our cohort the major part was diagnosed with stage 1 (60.6%). Most of the tumors in our cohort were Luminal-A-like or Luminal-B-like (HER2 negative) which is consistent to the data of Partridge et al. [24].

We were able to show a significant increase of SLNB over time then stable at a high level at 50–60% from 2010 onwards, which has also been shown by Schrodi et al. [27]. This is more likely due to the fact that SLNB was implemented as a standard procedure in the German guideline in 2008 [28], which was also the case in other countries such as the Netherlands [29, 30] and the USA [31].

Contrary to our analysis, other studies show that mastectomy rates have been increasing since the year of 2000 mainly in the US [32, 33]. In terms of treatment modalities, in our analysis young BC patients were more likely to undergo breast-conserving surgery (BCS), at least until 2012 with decreasing numbers in 2013 which then rise again slightly. The decreasing of BCS in our cohort is more likely due to the “Angelina-Jolie-Effect” in 2013 [34]. Generally, BCS reflects efforts to preserve breast aesthetics and function in the younger population, which may also be possible by the use of neoadjuvant chemotherapy. Body image concerns may be less for BCS patients, with or without reconstruction [35], which may explain findings of young women´s preferences for BCS, except when having children [36]. Randomised trials show no significant difference in survival benefits comparing modified radical mastectomy and BCS plus radiation [2, 3, 37, 38]. But it is also known that genetic predisposition and having children affect the preference for mastectomy [39, 40]. Additionally, as young age has been demonstrated as an independent risk factor for local recurrence after conservative treatment and more aggressive tumors in the younger age group, BCS is still under discussion [41–43]. However, the decision for breast conserving surgery versus mastectomy should be carefully weighed against each other, considering medical data such as tumor size, lymph node involvement, tumor biology, multifocality and patient preferences.

Additionally, our analysis highlighted that young BC patients receive aggressive treatment regimens: 43.2% received both chemotherapy and endocrine therapy, which is consistent to the literature [24]. Other studies could also show that the highest rate of chemotherapy can be found in patients younger than 40 years [24, 44, 45]. This underscores the challenges of managing BC in younger women, who may require intensified treatment strategies to achieve optimal outcomes as well as specifically addressed guidelines for treatment [46].

Notably, our survival analysis revealed favorable overall survival rates in young BC patients, particularly among those with Luminal A-like and Luminal-B-like tumors. The 5-year relative survival (RS) in our cohort was 90.9%, which is comparable to the literature [47]. But studies could show, that in luminal breast cancer, younger age (≤ 40 years) seems to be an independent prognostic factor [26, 48], but not in the more aggressive tumor phenotypes such as HER2-positive/non-luminal or triple-negative breast cancer compared with women 51–60 years of age [24]. This may reflect inadequate therapy, including lower treatment efficacy and less therapeutic adherence and persistence, as well as residual differences in tumor biology [24]. Tailored therapy in young patients seems to be an important step to reduce age-related disparities in breast cancer [24, 49].

We can confirm that regarding local recurrence in our cohort, Luminal-A-like and Luminal-B-like (HER2 negative) tumors were at a low risk, while HER2-positve/non- luminal and triple-negative breast cancer were at increased risk [50] of local recurrence as also a higher cumulative incidence of time to distant metastasis [46]. The poorer outcomes observed for triple-negative and HER2-positve/non luminal breast cancer remains consistent with other studies [50, 51] and highlight the need for effective systemic therapy in this important age group.

A limitation of this analysis is the possible underestimation of radiation therapy, endocrine therapy, and chemotherapy as a result of the inherent underreporting of therapies (particularly therapies not conducted, as well as those conducted but not reported) in the cancer registry. However, with the stepwise implementation of certified breast centers in Germany since 2006, data quality has increasingly improved. Furthermore, we have no information about menopausal state or personal data of the patient such as marital status, children, family planning, employment status. In addition, the law in Germany does not permit the collection and evaluation of data on genetic tests, which would have been interesting. Unfortunately, we cannot say from the available data who received which therapy and why, e.g. it cannot be determined retrospectively how many patients received SLNB+LAD, but not in how many patients with positive SLNB no LAD was performed.

## Conclusion

In conclusion, our analysis provides comprehensive insights into the clinicopathological characteristics, treatment patterns, and outcomes of BC in young women aged under 40 years. By elucidating the unique challenges and considerations associated with BC in this population, our findings contribute to the ongoing efforts to improve care and outcomes for young BC patients. Further research is warranted to better understand the underlying biological mechanisms driving BC in young women and to develop targeted therapeutic strategies tailored to their specific needs and preferences.

## Data Availability

No datasets were generated or analysed during the current study.
